# Implant-Based Chin Augmentation Vs Osseous Genioplasty: A Systematic Review of Indications and Outcomes

**DOI:** 10.1093/asjof/ojaf048

**Published:** 2025-06-26

**Authors:** Martin Kauke-Navarro, Leonard Knoedler, Omar Allam, Max Heiland, Samuel Knoedler, Felix J Klimitz, Michael Alperovich, Ali-Farid Safi

## Abstract

Osseous genioplasty (OG) and implant-based chin augmentation are 2 primary approaches for correcting microgenia. A comprehensive synthesis comparing their outcomes, safety profiles, and patient satisfaction is lacking. In this study, the authors aim to assess the safety and outcome profile of implant-based chin augmentation and OG. A systematic review was conducted following Preferred Reporting Items for Systematic Reviews and Meta-Analyses guidelines. A comprehensive search of PubMed, MEDLINE, Cochrane CENTRAL, and Google Scholar was performed in November 2024, utilizing keywords related to genioplasty, chin implants, and specific implant materials. Inclusion criteria required studies to provide original data on isolated procedures performed at a single institution, ensuring consistency in perioperative management. The authors included 7 single-center studies involving 1126 patients with microgenia (740 underwent OG; 386 received implants). Follow-up durations ranged from 6 months to 6 years. Implant materials included *Medpor*, silicone, *Proplast*, and PEEK. Patient satisfaction was high across both cohorts, with a trend toward higher satisfaction rates in the OG group. Complication profiles differed: implants had higher infection rates (up to 23.8%) and dehiscence, whereas OG was associated with transient neurosensory changes (up to 100%), occasionally persisting in 7.4% to 12.5% of patients. Relapse rates were similar but slightly higher for OG (2.63%-27.21%). OG was noted to provide greater soft-tissue predictability (85% bony to soft-tissue translation) than implants (66%). Both procedures are effective for managing microgenia, with distinct strengths and risks. Future studies should explore long-term outcomes and assess custom-fabricated implants to enhance fit, contour, and patient satisfaction. Tailored approaches based on individual patient needs remain critical.

**Level of Evidence: 3 (Therapeutic):**

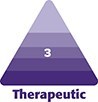

The anatomical and aesthetic significance of the chin in facial harmony has been well established, with chin hypoplasia—characterized by disproportionate mandibular projection relative to adjacent facial structures—representing a frequent indication for surgical intervention. The chin's role extends beyond mere aesthetic considerations, serving as the anatomical keystone that facilitates transition between facial and cervical planes, thereby fundamentally influencing overall facial balance.^1^ This anatomical relationship has particular relevance in masculine facial aesthetics, where chin prominence contributes significantly to gender-specific facial characteristics.^2^ Because of its significance, chin augmentation is one of the most commonly performed aesthetic facial skeletal augmentation procedures.^3^

Therefore, chin augmentation is a common aesthetic and reconstructive procedure aimed at enhancing facial harmony and correcting congenital or acquired chin hypoplasia.^4^ Although fillers and fat grafting are options to correct mild volume deficiencies, 2 primary techniques are used to correct moderate-to-severe volume deficiency: implant-based chin augmentation and osseous genioplasty (OG).^5^ Implant-based augmentation utilizes alloplastic materials to enhance chin projection, whereas OG involves controlled osteotomies to alter the position and contour of the mandibular symphysis for aesthetic and functional improvement.^6^ Although OG offers superior versatility in addressing complex geometric modifications and asymmetries, the relative technical simplicity of implant-based augmentation has led to its widespread adoption, particularly among surgeons without formal craniofacial training. In the current literature, there is a lack of articles that discuss the advantages, indications and spectrum of complications, and possibilities for either procedure.^7^

This review seeks to address this gap through detailed comparative analysis of implant-based augmentation and OG, with particular emphasis on establishing evidence-based guidelines for technique selection based on individual patient factors and desired outcomes.^8^ This review thus aims to fill this gap by comparing these 2 modalities, evaluating their indications, advantages, and disadvantages, and providing a detailed discussion on when each technique should be utilized.^8^

## METHODS

### Search Strategy

This systematic review was conducted in adherence to the Preferred Reporting Items for Systematic Reviews and Meta-Analyses (PRISMA) guidelines.^9^ A comprehensive search strategy was designed to identify relevant literature on OG and implant-based chin augmentation, focusing specifically on studies conducted in a single-center approach. The goal was to ensure consistent perioperative management and follow-up strategies between the 2 cohorts, thereby minimizing confounders associated with institutional variability.

**Figure 1.**
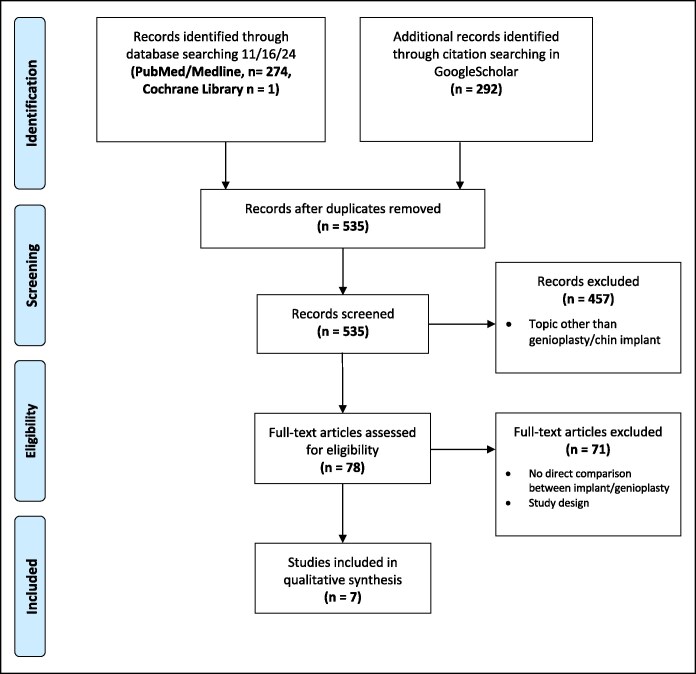
Preferred Reporting Items for Systematic Reviews and Meta-Analysis (PRISMA) flow diagram of study identification, screening, and inclusion.

The electronic databases PubMed, MEDLINE, Cochrane Central Register of Controlled Trials (CENTRAL), and Google Scholar were systematically searched in November 2024. The search terms included combinations of the following: “osseous genioplasty,” “sliding genioplasty,” “bony genioplasty,” “bone advancement genioplasty,” “osseous chin augmentation,” “chin implant,” “alloplastic augmentation,” and specific implant materials such as “*Medpor®*” “silicone,” “titanium,” “polyethylene,” and “Polyether ether ketone (PEEK).” Filters were applied to include only full-text articles written in English and involving human participants. The detailed search strategy and inclusion/exclusion process are illustrated in Figure 1 (PRISMA Flow Diagram).

For inclusion, articles had to present original data comparing outcomes of isolated OG and isolated implant-based chin augmentation in patients with similar indications, ensuring that these procedures were performed at the same institution. Additionally, studies were required to address outcomes related to retrognathia and to provide details on outcome profiles for both techniques. All articles in a language other than English were excluded. Studies discussing revision surgeries were excluded. Nonhuman studies, systematic reviews, meta-analyses, and articles lacking comparative data were excluded.

### Data Extraction

The detailed search strategy is shown in Figure 1 (Prisma flowchart). Two reviewers (M.K.-N. and A.-F.S.) independently assessed all articles, first by screening the title and abstract. Articles that met the inclusion criteria were then subjected to in-depth full-text review. Furthermore, all citations of the articles that were included were searched in Google Scholar. The data were extracted independently by 2 authors (M.K.-N. and A.-F.S.). The following variables were extracted, if available: year of publication, study type (eg, retrospective/prospective), number of patients, patient gender, duration of follow-up, operative time, implant type, type of genioplasty, indications for the procedure, complications (delay in wound healing, implant displacement, infection, hematoma/seroma, incidence of reoperation, neurosensory change, and outcome parameters), aesthetic outcomes, patient-reported outcomes, objective outcome variables, and additional procedural specifics. Because of the heterogeneity and small number of the included articles, a meta-analysis was not performed.

## RESULTS

### Study Characteristics

This review identified 7 single-center studies comparing outcomes of OG and implant-based chin augmentation, including a total of 1126 patients with microgenia (740 underwent OG and 386 received implants; Table 1). Most studies constituted retrospective cohort studies (6/7) with 1 prospective randomized controlled trial. Patient ages ranged from 15 to 50 years. The most prevalent implant material was porous polyethylene (Medpor, 4/7 studies), followed by silicone (2/7), polytetrafluoroethylene/Teflon (Proplast, 1/7), and PEEK (1/7). An overview of general study characteristics is summarized in Table 1, and a detailed summary of study characteristics is provided in Supplemental Table 1.^13-15,17-20^ Follow-up ranged from 6 months to 6 years (Table 2).

**Table 1.** General Study Characteristics and Surgical Details

Author (Year)	Study Design & N (Osteotomy/Implant)	Male (N)/Female (N)	Implant & Access	Genioplasty Bony Fixation	Implant Fixation
Tabrizi et al^19^ (2024)	Retrospective Cohort Study (38/42)	Genioplasty (29 F, 9 M) Implant (33 F, 9 M)	Medpor and Silicone Intraoral (19), Extraoral (23)	Chin plate and two lag screws	Two titanium screws (Medpor), Sutures (Silastic)
Guyuron and Raszewski^11^ (1990)	Retrospective Cohort Study (34/42)	Combined: 12 M, 64 F^a^	Proplast, Intraoral	Plates/Screws and wiring (pre-1986)	No fixation
Gui et al^12^ (2008)	Retrospective Cohort Study (500/150)	Genioplasty (90 M, 410 F) Implant (29 M, 121 F)	Medpor, Intraoral	Titanium plates and screws	Two titanium screws (6-8 mm, MedPor)
Bertossi et al^20^ (2015)	Retrospective Cohort Study (135/60)	Genioplasty (57 M, 78 F) Implant (22 M, 38 F)	Silicone, Extraoral	Three 0.6 mm titanium plates, 6 mm screws	No fixation (Silicone)
Mohammad et al^21^ (2010)	Retrospective Cohort Study (8/8)	Genioplasty (1 M, 7 F) Implant (3 M, 5 F)	Medpor, Intraoral	Titanium miniplates and screws	2 mm titanium screws (MedPor)
Helmy et al^22^ (2024)	Prospective randomized controlled (11/11)	Combined: 10% M, 90% F^a^	PEEK, Intraoral	Chin plat and two lag screws	Four titanium screws (PEEK)
Park et al^23^ (2010)	Retrospective Cohort Study (14/19)	Combined: 15 M, 18 F^a^	Medpor, Intraoral	Miniplates and screws	Miniscrews (MedPor)

N, Number; M, Male; F, Female. ^a^Gender breakdown by procedure type (genioplasty vs implant) was not reported in the original article.

**Table 2.** Complications and Patient Satisfaction

Author	Follow-up Duration	Outcome Assessment	Complications (N, %)
Tabrizi et al^19^	12 months	VAS score	Implant Displacement: None reported Infection: Genioplasty (2, 5.3%); Implant (10, 23.8%) Hematoma: None reported Reoperation: Genioplasty (1, 2.6%); Implant (8, 19%) Neurosensory Deficit: Genioplasty (13, 34.2%); Implant (8, 19%)
Guyuron and Raszewski^10^	Genioplasty (12 months), Implant (13 months)	Questionnaire, Postoperative discomfort on a scale from 1-5	Implant Displacement: None reported Infection: Genioplasty (0); Implant (2, 4.89%) Hematoma: None reported Reoperation: Implant (2, 4.89%), removal of implant due to infection Neurosensory Deficit: Temporary: Genioplasty (19, 70.4%); Implant (15, 46.9%) Sustained: Genioplasty (2, 7.4%); Implant (3, 9.4%)
Gui et al^8^	6 months to 6 years	Subjective assessment (patient/surgeon)	Implant Displacement: None reported Infection: N = 0 in both cohorts Hematoma: None reported Reoperation: Genioplasty (2, 0.4%) correction of contour irregularities at osteotomy sites Implant (2, 1.3%) needed revision to “reduce the implant’ Neurosensory Deficit: Genioplasty: Transient lip numbness in most patients; 1 patient (0.2%) with prolonged lower lip numbness >1 year Implant: None reported
Bertossi et al^20^	6 months to 3 years	Subjective assessment (patient/surgeon)	Implant Displacement: N = 3 (5%) Infection: N = 0 in both cohorts Hematoma: Genioplasty (12, 8%) requiring needle aspiration/compressive therapy Reoperation: Genioplasty (0), Implant (3, 5%) requiring implant removal and genioplasty Neurosensory Deficit: Temporary—Genioplasty (135, 100%)
Mohammad et al^21^	6 months	Subjective assessment (patient/surgeon)	Implant Displacement: N = 0 in both cohorts Infection: N = 0 in both cohorts Hematoma: N = 0 in both cohorts Reoperation: N = 0 in both cohorts Neurosensory Deficit: Genioplasty (1, 12.5%), temporary Implant (0)
Helmy et al^22^	6 months	FACE-Q	Implant Displacement: N = 0 in both cohorts Infection: N = 0 in both cohorts Hematoma: N = 0 in both cohorts Reoperation: N = 0 in both cohorts Neurosensory Deficit: Mild paresthesia of chin and lower lip that resolved within 2 weeks (unknown number of patients)
Park et al^23^	6 months	Subjective assessment (patient/surgeon)	No report on complications

### Procedural Details

Operative time was recorded in 1 article and was noted to be 35 min for OG and 20 min for implants.^21^ Details on operative approach and technique are summarized in Table 1. OG was consistently performed through an intraoral approach utilizing rigid fixation (titanium miniplates and screws). Implant placement access varied, with 5 studies reporting the use of an intraoral approach, whereas 1 study reported on the use of an extraoral approach. In 1 study, both approaches were reported in their cohort.^17^ For OG, all studies reported rigid fixation utilizing titanium plates and screws. Fixation in the setting of implant-based augmentation varied. *Medpor* and PEEK implants were secured with titanium screws in all studies.^16-18^ Silicone and Proplast implants were not fixated, with the exception of Tabrizi et al who used suture fixation of their silastic implants.^13,17,21^

### Complications

Complications were reported for both OG and implant-based chin augmentation, with notable differences in frequency and type. Dehiscence was primarily observed in the implant group, with rates up to 23.8%, compared with 5.3% in the OG group. Infections were also more prevalent in implant cases, ranging from 0% to 23.8%, whereas OG reported a lower range of 0% to 5.3%. Extraoral approaches were used in 2 studies: Tabrizi et al employed a mixed approach with both intraoral and extraoral placements, whereas Bertossi et al exclusively used an extraoral approach.^13,17^ Infection rates varied considerably between studies and surgical techniques. Tabrizi et al reported an overall infection rate of 24%, with a significant difference between intraorally placed implants (42%) and extraorally placed implants (8.7%).^17^ In contrast, Bertossi et al reported no infections (0%) in their extraoral implant group.^13^ Guyuron and Raszewski noted a 5% infection rate for intraorally placed implants.^21^ Infections were rarely observed in the genioplasty cohort, with only 2 cases reported across all studies. Hematomas were exclusively documented in the OG group in 1 study.

Neurosensory changes were a common complication in the OG group, noted in 6 out of 7 studies. Temporary neurosensory deficits were reported in up to 100% of patients, with most resolving over time.^13,14,17,21^ However, prolonged neurosensory disturbances lasting beyond 1 year were observed in 7.4% to 12.5% of patients, as described in 2 studies.^19,21^ In contrast, temporary neurosensory changes in the implant cohort were reported in only 2 studies, with rates ranging from 19% to 46.95% of patients.^17,21^ Persistent neurosensory deficits were noted in 1 study in the implant cohort (9.4%).^21^

Reoperation rates were higher for implants, reported in 4 studies, with a range of 0% to 19% of patients in a single study requiring return to the operating room. In comparison, reoperation rates for OG ranged from 0% to 2.6% and were reported in 3 studies. The highest reoperation rates were seen in studies reporting wound dehiscence and infections. Tabrizi et al documented a 19% reoperation rate for implants, with 24% of cases experiencing dehiscence (all implants that were placed through an intraoral incision).^17^ Guyuron and Raszewski reported the removal of 2 implants because of infection.^21^ Bertossi et al noted the removal of 3 implants because of displacement.^13^ Implant displacement was generally rare and only reported in 1 study by Bertossi et al who used silicone implants.^13^ In the majority of implant cases (*Medpor*, PEEK, Silicone), the implants were secured with either sutures or rigid fixation methods such as titanium screws.^14-17,19^

### Outcomes

#### Patient Satisfaction

Patient satisfaction was evaluated in most studies, although the methods varied widely and were often inconsistent, with some studies relying solely on subjective outcome assessments. Tabrizi et al utilized a Visual Analog Scale (VAS) to score satisfaction from 0 to 10, categorizing scores as follows: 0 to 3 (dissatisfied), 4 to 6 (satisfied), and 7 to 10 (highly satisfied).^17^ On average, patients undergoing OG (mean, 7.8) had higher VAS values when compared with those undergoing implant-based chin augmentation (mean, 6.6-6.7).

Guyuron and Raszewski implemented a custom-designed, patient-directed questionnaire to evaluate outcomes that included questions on patient comfort, satisfaction, and overall satisfaction with the procedure.^21^ Both OG and implant-based augmentation showed similar results, with higher satisfaction and improvement in self-esteem in the OG group. Postoperative discomfort was noted to be higher in the OG group.^21^

Gui et al relied exclusively on subjective assessments, whereas Bertossi et al and Park et al did not provide data on any measures of patient satisfaction.^13,16,19^ Mohammad et al provided subjective assessments of both outcomes and satisfaction.^14^ In contrast, Helmy et al employed the validated FACE-Q tool to evaluate postoperative outcomes. According to Helmy et al, all patients were satisfied, and there was no significant difference between the OG and implant groups. Detailed results are summarized in Table 2, whereas a comparison of general characteristics of implant-based chin augmentation and OG is shown in Table 3.

**Table 3.** Comparison of General Characteristics of Implant-Based Chin Augmentation and Osseous Genioplasty

Category	Implant-Based Chin Augmentation	Osseous Genioplasty
Common Indications	Mild/moderate chin deficiency^21^	Most cases of microgenia, specifically for severe chin deficiency^24^
	Patient preference for less invasive procedure^19^	3D corrections
	Adequate soft tissue coverage	Previous implant failure and salvage after implant placement^24,25^
	Adjunct to other procedures (eg, face lift/rhinoplasty)^26^	
Advantages	Less invasive, faster procedure^10^ Extraoral placement^27^	Comprehensive correction Can improve cervicomental angle, can increase airway space^10,15,19^
	Reversible	Natural results
	Immediate results	Long-term stability
	Option of 3D printed/custom made implants^28-30^	More versatile^24,31^
	Higher Soft Tissue Response to Hard Tissue Movement^10^	Option of using custom made cutting guides and plates^8,32^
Disadvantages	Higher risk of complications regardinginfection, extrusion, foreign body reactions, skin ulcerations, bony chin erosion^33-37^	Higher complication risks regarding nerve injury, malunion^34^ More invasive procedure, more perioperative discomfort^10^
	Limited corrections for severe deformities Unable to augment vertically^27^	Requires advanced surgical skills
	Longevity issues (potential need for revision)	
	Bone resorption, migration, lower patient satisfaction^31^	
Ideal Candidates	Patients seeking minimal invasiveness and quick recovery	Patients needing extensive correction and stability
	Patients with mild to moderate deficiencies	Patients with severe deficiencies or complex asymmetries
		Patients with prior implant complications
Typical Complications	Infection, extrusion, bone resorption^24,25,35,39^	Nerve injury (at least 10%), malunion, nonunion^40,42^
Recovery Time	Shorter^38^	Longer
Predictability	High for mild to moderate corrections	High for severe and complex corrections
Surgical Time	Generally shorter^10^	Generally, longer
Postoperative Discomfort	Less^8,38^	More
Equipment	No special equipment needed^24^	Special equipment needed to perform osteotomies (eg, reciprocating saw)

#### Soft-Tissue Changes and Relapse Rates

Three studies examined soft-tissue changes and relapse rates in both the OG and implant cohorts (see Supplemental Table 2 for details).^13,14,16^ Soft-tissue changes in all 3 studies were assessed utilizing lateral cephalograms and measuring the soft-tissue pogonion (Pog′) distance to a true vertical line. Advancement ranged from 4.89 to 12.13 mm in the OG cohort and from 5.6 to 11.25 in the implant cohort. Sliding genioplasty showed relapse rates ranging from 2.63% to 27.21%. Implants, including silicone and *Medpor*, demonstrated slightly lower relapse rates (5.36%-25.07%) and better soft-tissue stability in some cases. Relapse was highest in those patients who showed the greatest pre- to postoperative advancement.^14^ Changes were statistically significant in the study performed by Park et al, demonstrating a relapse rate of 18.59% in the OG cohort vs 14.56% in the implant cohort.^16^

Furthermore, Guyuron and Raszewski found that the hard tissue to soft-tissue response rate was higher in the OG cohort (85% of bony advancement was reflected in soft-tissue change), whereas the number was at 66% in the implant cohort.^21^ Helmy et al discussed soft-tissue changes in both cohorts. However, reporting was inconsistent and, therefore, not included in the synthesis. They concluded that the relapse rate was similar between both groups at about 8% to 14%.

## DISCUSSION

Microgenia is the most common form of chin deformity, as described by Guyuron et al.^18^ It is typically classified as mild, moderate, or severe based on standardized cephalometric landmarks, although gender- and ethnicity-specific features influence these assessments.^41^ A published classification system utilizes the horizontal distance between pogonion and the zero-meridian line, a vertical line extending from Nasion perpendicular to the Frankfort horizontal plane, as originally defined by González-Ulloa.^41,42^ Ideally, pogonion should align with this meridian. Based on the original work by González-Ulloa, mild microgenia was defined as a retraction of <10 mm, moderate as 10 to 20 mm, and severe as >20 mm.^41^

In this review, we found that implants appear to provide stable and satisfactory outcomes in patients with mild-to-moderate chin deficiencies (mainly to correct sagittal deficiency at the pogonion and width deficiencies at the level of the symphysis) with less postoperative discomfort and faster recovery.^13,17^ In cases of severe chin hypoplasia, both procedures can be combined to achieve adequate volumetric augmentation and considerable advancements.^14^ Although implant-based chin augmentation in cases of mild retrogenia (no previous surgical intervention) is generally accepted, OG may have several advantages, such as versatility in movements and correction of complex 3-dimensional (3D) deformity.^8^ One of the more relevant advantages is the functional effect of chin advancement, such as an increase in airway space, which cannot be achieved by implant placement.^26^

In terms of patient satisfaction, outcomes were overall comparable between the 2 cohorts and patients were generally satisfied.^13-15,17,19,21^ We identified variable methods of assessing patient satisfaction, which limits the ability to compare results between studies. Nevertheless, across all studies, both implant-based chin augmentation and OG were able to achieve satisfactory patient-reported outcomes without significant differences, as reported by Helmy et al, Darwich et al, and Gui et al^15,16,19^ In 2 studies with detailed outcome assessment, a trend was noted that OG generally leads to higher patient satisfaction rates.^17,21^

The comparative safety profiles of these procedures reveal distinct patterns of complications specific to each approach. Our analysis demonstrates that although both techniques maintain acceptable overall safety profiles, they present procedure-specific risks that warrant careful consideration during surgical planning. Implant-based augmentation demonstrates a notably higher susceptibility to infectious complications, with infection rates reaching 23.8% in some series. These infections frequently necessitate implant removal, contributing to elevated reoperation rates in this cohort. This stands in marked contrast to the OG group, where infection rates typically range from 0% to 5.3%. The higher infection risk in implant cases appears primarily attributable to the presence of alloplastic material, with additional reoperations often required to address both infectious complications and contour irregularities.^17^ Conversely, neurosensory disturbances show a different distribution pattern between the 2 techniques. OG is associated with a significantly higher incidence of neurosensory changes, affecting up to 100% of patients in the immediate postoperative period. Although most of these sensory alterations prove temporary, several studies document rates of permanent nerve dysfunction ranging from 7.4% to 12.5%. This contrasts sharply with implant-based procedures, which demonstrate both a lower rate of temporary sensory changes and a markedly reduced incidence of permanent neurologic sequelae.

Soft-tissue change stability and relapse rates were assessed in 3 of the studies. Across the 3 studies, there was a trend toward a higher relapse rate in OG (2.63%-27.21%) compared with implants (5.36%-25.07%). On the other hand, Guyuron and Raszewski discussed hard tissue response rates, noting that 85% of bony advancement in OG is reflected in soft-tissue projection, compared with only 66% of augmentation in the implant cohort.^21^ Although specific measurements were not provided, this finding suggests that OG may offer greater predictability and stability in achieving desired aesthetic outcomes in the short term. Further, the data provided by Bertossi et al, Mohammad et al, and Park et al suggest that with larger advancements, the soft-tissue relapse rate may be higher in the OG cohort.^13,14,16^ This phenomenon may be attributed to bone resorption following remodeling in cases of large advancement genioplasties. Polido et al demonstrated that significant advancements (minimum 8 mm, with an average of 11.7 mm) resulted in a predictable long-term resorption of ∼24% of the initial advancement after a minimum follow-up of 6 months (genial bone resorption) and soft-tissue changes during healing.^17^ In the studies included herein, Mohammad et al reported high relapse rates associated with larger advancements (mean chin movement of 10.75 mm), which aligns with findings from previous literature and similar reports.^14^

In the implant cohort, relapse may be because of insufficient soft-tissue adaptation or integration over time. Additionally, because measurements in the implant group were taken immediately postoperatively, initial edema may have inflated soft-tissue projection, with subsequent resolution over time contributing to a perceived “relapse.” Subperiosteal resorption was not reported in any of the studies for implant patients, likely because of the short follow-up period. However, this remains a well-documented long-term complication, particularly in patients with silicone implants, and could potentially influence long-term outcomes, which are still poorly understood.^6,7^

Lastly, the analyzed studies demonstrated a predominance of 2 implants: *Medpor*, employed in 4 of 7 studies, and silicone in 2, reflecting contemporary practice in chin augmentation procedures. The literature widely recognizes silicone and *Medpor* implants as the most commonly used chin augmentation materials, both demonstrating generally safe risk profiles. In terms of implant choice, silicone implants are easy to place and remove but have higher risks of infection, displacement, and subperiosteal resorption, particularly when placed intraorally.^6,17,18^ In contrast, *Medpor* implants integrate with surrounding tissue, reducing displacement risk and improving stability but making removal more challenging if complications occur.^18^ Although *Medpor* implants may have lower infection rates, their rigid fixation often requires screws, and long-term outcomes for both materials remain insufficiently studied.^7,18^

Notably, all investigations utilized commercial implants without exploring custom-fabricated alternatives. This standardized approach presents inherent limitations in achieving optimal facial contours, particularly evident with silicone implants, which preclude intraoperative modifications for enhanced fit and symmetry. The current reliance on off-the-shelf implants suggests an opportunity for technological advancement in the field. Modern 3D imaging and computer-aided manufacturing capabilities could facilitate the development of patient-specific implants based on precise preoperative facial analysis. Such customization might address current limitations in implant conformity while potentially optimizing aesthetic outcomes and patient satisfaction. To be sure, the implementation of custom implant technology necessitates careful evaluation of the cost–benefit relationship, considering increased manufacturing complexity and associated expenses. Future research protocols should incorporate standardized outcome measures and extended follow-up periods to assess whether the presumed benefits of patient-specific designs translate into clinically meaningful improvements that justify additional resource allocation.^6,27,28^

In summary, future studies should focus on long-term outcomes, standardizing satisfaction assessment, and exploring custom-fabricated implants to address fit and contour limitations, ultimately improving patient outcomes and satisfaction.

### Limitations

Several limitations are inherent to the methodology and available studies. The articles did not specify their approach to determining eligibility for either OG or implant-based augmentation, and little is known about preoperative assessment and aesthetic analysis (eg, chin pad analysis and labiomental fold analysis) and how this determined which procedure the patient underwent. Hence, this review is limited by potential preselection bias, as patients with milder deformities were more likely to receive implant-based augmentation, whereas OG was often reserved for more complex skeletal movements, potentially influencing outcome comparisons. Outcome reporting was inconsistent between studies, and follow-up was generally short. Furthermore, we noted a predominance of retrospective cohort studies (6/7) and regional variability (eg, the United States, China, Italy, and South Korea), which may limit the generalizability of results and introduce regional differences. Lastly, the experience and skill of the surgeon play a critical role in outcomes, but this variable could not be accounted for in this systematic review.

## CONCLUSIONS

This review highlights that both OG and implant-based chin augmentation achieve high patient satisfaction, with a trend toward higher satisfaction in the OG cohort. Complications varied, with implants having higher risks of infection and dehiscence, whereas genioplasty was associated with transient neurosensory changes. Relapse rates were comparable, although OG showed greater soft-tissue predictability, with more bony advancement translating into soft-tissue projection. Implant-based approaches demonstrated better long-term stability in some cases.

## Supplemental Material

This article contains supplemental material located online at https://doi.org/10.1093/asjof/ojaf048.

## Supplementary Material

ojaf048_Supplementary_Data
