# Supplementary material for: Implant-Based Chin Augmentation Vs Osseous Genioplasty: A Systematic Review of Indications and Outcomes
Source: Aesthet Surg J Open Forum. 2025 Jun 26;7:ojaf048. doi: 10.1093/asjof/ojaf048 (PMC12262119; doi:10.1093/asjof/ojaf048)
Supplement: ojaf048_Supplementary_Data [file ojaf048_supplementary_data.docx]

**Table S1.** Detailed overview of study characteristics and surgical details.

| **Author (Year)** | **Country** | **Study Design** | **Sample Size (Osteotomy/ Implant)** | **Patient Age (y, Mean ± SD)** | **Implant Type** | **Male (n) / Female (n)** | **Genioplasty Bony Fixation** |  | **Implant Fixation** | **Indication for procedure and recommendations** | **Genioplasty: Mean chin movement (mm)** | **Access for implant placement, n** |
| --- | --- | --- | --- | --- | --- | --- | --- | --- | --- | --- | --- | --- |
| Tabrizi et al.^1^ (2024) | Iran | Retrospective Cohort Study | 38 / 42 | Genioplasty (30.6 ± 6.5), Implant (30.59 ± 7.16) | Medpor and Silicone | Genioplasty (29 F, 9 M) Implant (33 F, 9 M) | Chin plate and two lag screws |  | Two titanium screws ( Medpor), Sutures (silastic) | Osteotomy and Implant: “Retrognathia” | NA | Intraoral (19), Extraoral (23) |
| Guyuron & Raszewski^2^ (1990) | USA | Retrospective Cohort Study | 34 / 42 | Genioplasty (30.85), Implant (42.37) | Proplast | 12 Male / 64 Female | Plates/Screws and wiring (pre-1986) |  | No fixation | Microgenia  Osteotomy: Severe cases, marked retrogenia or retrognathia, asymmetries  Implant: Older patients (>50), proper cervcomental angle. | NA | Intraoral (42) |
| Gui et al.^3^ (2008) | China | Retrospective Cohort Study | 500 / 150 | Genioplasty (Range: 18-45)  Implant (Range: 18-40) | Medpor | Genioplasty (90 M, 410 F)  Implant (29 M, 121 F) | Titanium plates and screws |  | Two titanium screws (6-8 mm, MedPor) | Genioplasty: most patients, especially severe cases  Implant: mild to modest retrogenia and minor contour irregularities | 6.5 horizontally, 5.5 vertically | Intraoral (150) |
| Bertossi et al.^4^ (2015) | Italy | Retrospective Cohort Study | 135 / 60 | For all patients (34.5) | Silicone | Genioplasty (57 M, 78 F) Implant (22M, 38 F) | Three 0.6 mm titanium plates, 6 mm screws |  | No fixation (Silicone) | Osteotomy and Implant: “Chin microgenia”  Osteotomy: severe microgenia and retrogenia, asymmetry, syndromic  Implant: Mild retrogenia | Osteotomy: In 135 patients’ sagittal movement 7-8 mm  In 34 patients: 3-5 mm vertical augmentation  Implant: | Extraoral (60) |
| Mohammad et al.^5^ (2010) | India | Retrospective Cohort Study | 8 / 8 | For all patients age range 15-37 | Medpor | Genioplasty (1 M, 7 F) Implant (3 M, 5 F) | Titanium miniplates and screws |  | 2 mm titanium screws (MedPor) | Implant: mild to moderate horizontal chin deficiency, minor contour irregularities  Genioplasty: “any chin” | Mean horizontal chi movement 10.75 mm, for implant mean chin augmentation 8.5 mm | Intraoral (8) |
| Helmy et al.^6^ (2024) | Egypt | Prospective randomized controlled | 11 / 11 | For all patients (25.5±3.5) | PEEK | 10% male/ 90% female | Chin plat and two lag screws |  | Four titanium screws (PEEK) | Genioplasty and Implant: Patients with “deficient chin with mild retrogenia” | NA | Intraoral (11/11) |
| Park et al.^7^ (2010) | South Korea | Retrospective Cohort Study | 14/19 | For all patients 22 years, range 18-37 | Medpor | 15 Male/ 18 Female | Miniplates and screws |  | Miniscrews (MedPor) | For both: “Patients requiring chin augmentation” | Genioplasty: Horizontal movement: 4.49 ± 1.78  Implant: 7.05 ± 6.21 | Intraoral (19/19) |

N = Number; M = Male; F = Female; SD = Standard De

**Table S2.** Reported Soft Tissue Changes and Relapse rates in 3 studies.

| **Study Name** | **Procedure** | **Start Value (Postoperative, mm) Time point** | **End Value** | **Relapse (mm)** | **Percent Relapse** | **Time Points** | **Measurement Method** |
| --- | --- | --- | --- | --- | --- | --- | --- |
| **Bertossi et al.** | Sliding Genioplasty | 7.6 (“immediately postoperatively”) | 7.4 (3 years) | 0.2 | 2.63% | Post-op, 3 years | Lateral Cephalograms, Pog′ measured relative to the True Vertical Line (TVL), parallel to the Frankfort Horizontal (FH). |
|  | Silicone Implant | 5.6 (“immediately postoperatively”) | 5.3 (3 years) | 0.3 | 5.36% | Post-op, 3 years | Pog′ measured relative to the TVL; more soft tissue adaptation noted with the implant. |
| **Mohammad et al.** | Sliding Genioplasty | 12.13 (1 week) | 8.83 (6 months) | 3.3 | 27.21% | 1 week, 6 months | Lateral Cephalograms, Pog′ changes measured relative to the True Vertical Line (TVL), perpendicular to a horizontal plane oriented 7° inferior to the Sella-Nasion plane. |
|  | Medpor Implant Augmentation | 11.25 (1 week) | 8.43 (6 months) | 2.82 | 25.07% | 1 week, 6 months | Same as above |
| **Park et al.** | Sliding Genioplasty | 4.89 (“postoperative”) | 4.01 (6 months) | 0.88 | 18.59% | Pre-op, post-op, 6 months | Lateral cephalograms; Pog′ measured relative to a line parallel to a modified Sella-Nasion plane. |
|  | Medpor Implant | 6.96 (“postoperative”) | 6.12 (6 months) | 0.84 | 14.56% | Pre-op, post-op, 6 months | Same methodology: showed smaller relapse compared to genioplasty. |
